# Complete Genome Sequence of the *Clostridium difficile* Type Strain DSM 1296^T^

**DOI:** 10.1128/genomeA.01186-15

**Published:** 2015-10-08

**Authors:** Thomas Riedel, Boyke Bunk, Johannes Wittmann, Andrea Thürmer, Cathrin Spröer, Sabine Gronow, Heiko Liesegang, Rolf Daniel, Jörg Overmann

**Affiliations:** a Leibniz Institute DSMZ-German Collection of Microorganisms and Cell Cultures, Braunschweig, Germany; b German Centre of Infection Research (DZIF), Partner site Hannover-Braunschweig, Braunschweig, Germany; c Department of Genomics and Applied Microbiology and Göttingen Genomics Laboratory, Georg-August-University, Göttingen, Germany

## Abstract

In this study, we sequenced the complete genome of the *Clostridium difficile* type strain DSM 1296^T^. A combination of single-molecule real-time (SMRT) and Illumina sequencing technology revealed the presence of one chromosome and two extrachromosomal elements, the bacteriophage phiCDIF1296T and a putative plasmid-like structure harboring genes of another bacteriophage.

## GENOME ANNOUNCEMENT

*Clostridium difficile* infections have conspicuously increased in recent decades and are among the most prevalent and costly infectious disease worldwide. The bacterium was first described in 1935 as part of the intestinal flora of neonates (1, 2). In this study, we determined *de novo* the complete genome sequence of the *C. difficile* type strain DSM 1296^T^ (=ATCC 9689^T^) using a combination of single-molecule real-time (SMRT) and Illumina sequencing technology.

*C. difficile* strain DSM 1296^T^ was cultivated anaerobically in Wilkins-Chalgren Anaerobe Broth (Oxoid, Basingstoke, United Kingdom) at 37°C. Genomic DNA was extracted using the Genomic-tip 100/G kit (Qiagen, Venlo, the Netherlands), according to the instructions of the manufacturer, with the following modification: directly after 1 h of lysis with lysozyme, 0.5 M EDTA (pH 8.0) was added to a final concentration of 20 mM, followed by incubation with proteinase K overnight.

Genome sequencing of strain DSM 1296^T^ was carried out on the PacBio *RSII* (Pacific Biosciences, Menlo Park, CA) using P5 chemistry. Genome assembly was performed with the RS_HGAP_Assembly.3 protocol included in SMRT Portal version 2.2.0, utilizing 56,696 postfiltered reads, with an average read length of 9,898 bp. Three contigs were obtained that represented three different replicons. Each contig was trimmed and circularized. In parallel, genome sequencing was carried out on a GAIIx Genome Analyzer (Illumina, San Francisco, CA) in a 112-bp paired-end single-indexed run, resulting in 3.8 million paired-end reads. Quality improvement of the final consensus sequence was performed with the Burrows-Wheeler Aligner (BWA) (3), mapping the Illumina reads onto the contigs obtained through PacBio sequencing. This approach yielded a final quality score of QV60. Automated genome annotation was carried out using Prokka (4).

The chromosome of *C. difficile* DSM 1296^T^ has a size of 4,109,692 bp and contains 3,596 predicted coding sequences, 35 rRNAs, and 90 tRNAs. The G+C content is 28.8%. Further analysis of the chromosome revealed the presence of genes encoding the complete pathogenicity locus associated with toxin production (CDIF1296T_00820 to CDIF1296T_00825). In addition, the genes encoding a Wood-Ljungdahl pathway cluster (CDIF1296T_00886 to CDIF1296T_00900), a carbon monoxide dehydrogenase (CODH) cluster (CDIF1296T_00296 to CDIF1296T_00298), a ferredoxin:NAD^+^-oxidoreductase (RNF) complex (CDIF1296T_01211 to CDIF1296T_01216), formate dehydrogenases (CDIF1296T_00938/CDIF1296T_02282/CDIF1296T_03435), and hydrogenases (CDIF1296T_01059/CDIF1296T_01060/CDIF1296T_03372/CDIF1296T_03512 to CDIF1296T_03514) were detected (5, 6). Also, mobile elements, such as transposons (e.g., CDIF1296T_01877 to CDIF1296T_01907) and prophages (e.g., CDIF1296T_00320 to CDIF1296T_00407) were detected.

As part of the genome, two additional replicons were identified, including the recently published novel temperate *C. difficile*-infecting bacteriophage phiCDIF1296T (7) and a putative plasmid-like structure of 45,187 bp that carries gene clusters that are typical for bacteriophages. These gene clusters of the latter replicon encode proteins for head and tail structures, host cell lysis, or replication. We identified genes for a putative terminase (CDIF1296T_03872), several tail and head proteins (e.g., CDIF1296T_03824 and CDIF1296T_03875), an integrase (CDIF1296T_03870), a helicase (CDIF1296T_03858), a single-stranded binding (Ssb)-like protein (CDIF1296T_03862), and an amidase (CDIF1296T_03832). Furthermore, genes for a ParM-like protein (CDIF1296T_03837) and a RepB-like protein (CDIF1296T_03844) were detected.

### Nucleotide sequence accession numbers.

The nucleotide sequence has been deposited at GenBank under the accession numbers CP011968 and CP011969. The versions described in this paper are versions CP011968.1 and CP011969.1.
